# Influence of 4% icodextrin solution on peritoneal tissue response and adhesion formation

**DOI:** 10.1186/1471-2482-13-34

**Published:** 2013-09-10

**Authors:** Christian D Klink, Patrick Schickhaus, Marcel Binnebösel, Stefan Jockenhoevel, Rafael Rosch, Rene Tolba, Ulf P Neumann, Uwe Klinge

**Affiliations:** 1 Department of General, Visceral and Transplantation Surgery, University Hospital Aachen, Pauwelsstr 30, 52074 Aachen, Germany; 2 Department of Tissue Engineering & Textile Implants, Applied Medical Engineering, Helmholtz Institute for Biomedical Engineering, RWTH Aachen University, Aachen, Germany; 3 Institute for Laboratory Animal Science, RWTH Aachen University Hospital, Aachen, Germany

**Keywords:** Postoperative peritoneal adhesions, Icodextrin, Adept, Inflammatory response, Peritoneal wound healing

## Abstract

**Background:**

Postoperative peritoneal adhesion formation following abdominal surgery remains a relevant surgical problem. The application of soluble physico-chemical barriers like 4% icodextrin is one approach to protect the peritoneal surface from getting linked to adhesive scar. The aim of this study was to investigate the influence of 4% icodextrin on peritoneal tissue response both of visceral and parietal peritoneum, adhesion formation and wound healing.

**Methods:**

40 rats were divided into two groups. After creation of an intraabdominal defect, either 4% icodextrin (Adept®) or sodium chloride was applied. Animals were sacrificed after 7 and 21 days. Adhesions were scored by an adhesion score. Furthermore, immunohistochemical investigations were conducted to determine the discrete influence of icodextrin on the parietal and visceral peritoneal tissue responses (CD68^+^ macrophages, CD3^+^ T-lymphocytes, vimentin for mesenchymal cells, HBME-1 for mesothelial cells, and as components of wound healing COX-2, C-myc, catenin).

**Results:**

Postoperative peritoneal adhesions were predominantly present in the sodium chloride group as compared to the icodextrin group (14/19 (74%) vs. 9/19 (47%); p = 0.048). The adhesion score however did not reveal any significant differences, (p = 0.614). Furthermore, the expression of vimentin in both the parietal and visceral peritoneum after 21 days was significantly lower in the icodextrin group than in the sodium chloride group (p = 0.038 and p = 0.028, respectively). No significant differences were observed for macrophages, lymphocytes, reperitonealisation or the expression of COX-2, C-myc or Catenin.

**Conclusions:**

The intraperitoneal application of 4% icodextrin reduces adhesion formation in comparison to sodium chloride. 4% icodextrin solution reduces the inflammatory and mesenchymal infiltrate in the wounded area, thus improving the ratio of mesothel cells to mesenchymal infiltrate. As demonstrated, icodextrin is able to ameliorate the local tissue response. Further experimental studies would be done to elaborate the impact on the early response of the adaptive immune system, which may then trigger the subsequent wound healing and tissue repair.

## Background

The formation of postoperative peritoneal adhesions after abdominal surgery remains a major surgical problem causing complications like obstipation and ileus [1-3]. Peritoneal adhesion rates higher than 90% after abdominal surgery have previously been reported [4,5]. Intraabdominal application of various substances including hyaluronic acid/carboxymethylcellulose and 4% icodextrin solution (Adept®) or systemic application including dimetindene maleate were used in order to reduce peritoneal adhesions [2,6]. Although in 2007 *Brown et al.* showed significantly reduced adhesion formation of icodextrin in comparison to lactated Ringer’s solution [7], the effect of icodextrin remains controversial. *Kumar et al.* demonstrated that icodextrin has a reductive influence on adhesion formation, but not on consecutive complications [8]. Recently, *Catena et al.* launched the first prospective, randomized controlled investigation regarding the influence of icodextrin on adhesion formation [9]. The intraabdominal application of icodextrin permits an even distribution. It is assumed to be preserved for 3–5 days after surgery, during the time of highest risk for adhesion appearance [10]. Tissue surfaces are kept apart by flotation offering a sufficient barrier for adhesion formation [11-13]. So far, no local or systemic side-effects have been described during metabolism and degradation of icodextrin [12,14,15].

Defects of appropriate repair mechanisms and persistent inflammatory processes have been described as potential reasons for adhesion formation [16-18]. The aim of this study was to determine the impact of icodextrin on the local tissue response of visceral and parietal peritoneum in comparison to sodium chloride in a rat model, and to analyse the potential inflammatory response markers (CD68, CD3 and COX-2), wound healing (C-myc, catenin), mesothelium (mesothelial cells) regeneration and cell integrity (vimentin).

## Methods

The experiments were officially approved by the local Animal Care and Use Review Committee (Landesamt für Natur, Umwelt und Verbraucherschutz Nordrhein-Westfalen, AZ8.87-50.10.35.08.319). All animals received humane care in accordance with the requirements of the German Animal protection Law, §8 Abs. 1 and in accordance with the Guide for the Care and Use of Laboratory Animals published by the National Institute of Health.

### Animals

40 male Wistar rats with a mean bodyweight of 200–300 g were randomly divided into two groups consisting of the icodextrin group (*n* = 20) and the sodium chloride group (*n* = 20). All animals were kept under standardized conditions: temperature between 22°C and 24°C; relative humidity 50-60%; 12 h of light following 12 h of darkness. The animals had free access to food and water. Food was withdrawn 12 h before and after surgery. All operations were carried out under general anesthesia and aseptic and sterile surgical conditions.

### Surgical procedure

**Figure 1:**
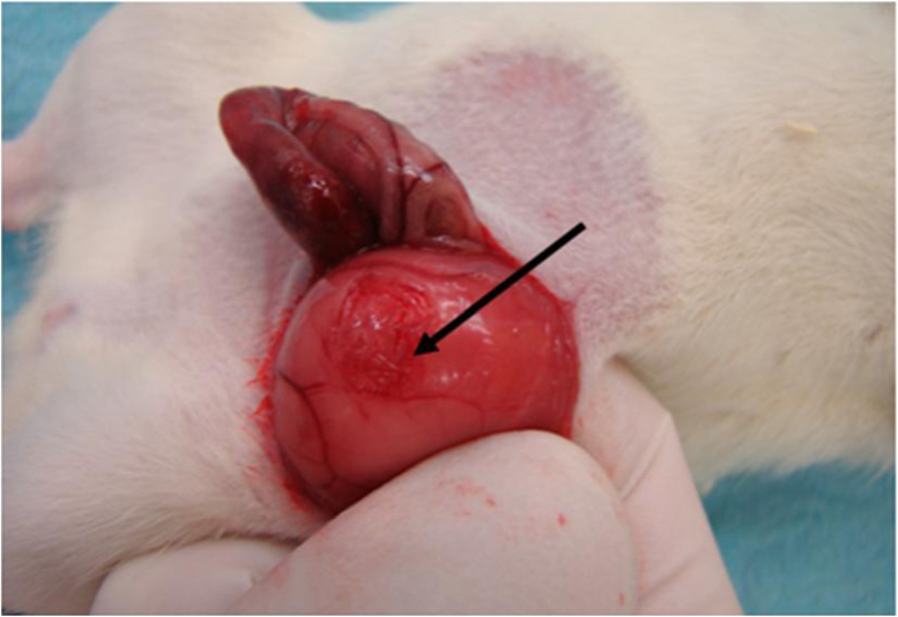
1 cm^2^ standardised defect zone by scalpel in the parietal peritoneum of the lateral abdominal wall.

Operations were carried out under general anesthesia. General anesthesia was achieved with a subcutaneous mixture of 0.3 mg/kg medetomidine (Domitor®, Pfizer AG, Zurich, Switzerland) and ketamine hydrochloride 100 mg/kg (Ketamin® 10%, Sanofi-Ceva, Düsseldorf, Germany). A 6 cm midline incision was performed. After laparotomy, a 1 cm^2^ standardized defect was created in the parietal peritoneum of the left lateral abdominal wall (Figure 1) using a scalpel. Visceral adhesion induction was performed under sterile conditions using a standardized abrasion model as previously described (5 cm proximal of the ileocoecal valve) [10]. The subjects were randomly placed into the different treatment groups, and either 10 ml/kg of icodextrin 4% (Adept®, Baxter, USA) or 10 ml/kg of sodium chloride was administered intraperitoneally. Dosage was assumed from prior publication [19]. Closure of the fascia was performed with 3/0 polyglactin continuous sutures (Vicryl®, Ethicon Inc., Somerville, NJ, USA). Skin closure was subsequently done with 4/0 polypropylene (Prolene®, Ethicon Inc., Somerville, NJ, USA) single sutures. No additional antibiotic treatment was given before or during the experiments. Throughout the whole observation period, all animals underwent daily clinical investigation to assess local and systemic complications. For exact quantitative measurement of adhesions, a post mortem examination was performed. The abdominal cavity was opened using a U-shaped incision, with its base in the lower abdomen for complete exploration. The presence of adhesion was registered. Adhesion score was performed (see below) and tissue specimens were obtained from the visceral and the parietal defect in the presence of adhesions, for further immunohistochemical investigations.

### Adhesion score

Postoperative peritoneal adhesions were scored by observers who were blinded to the study groups using an established adhesion score published by *Diamond et al.*, based on extent, type, and tenacity of the adhesion tissue both for visceral and parietal peritoneum (Table 1) [20].

**Table 1:** **Adhesion score by Diamond et al. [**[20]**]**

	**Score**
**Extent**	
- 0%	0
- < 25%	1
- <50%	2
- <75%	3
- >75%	4
**Type**	
- None	0
- Filmy, no vessels	1
- Opaque, no vessels	2
- Opaque, small vessles	3
- Opaque, large vessels	4
**Tenacy**	
- None	0
- Easily lysed	1
- Lysed with traction	2
- Required sharp dissection	3
**Total score**	**11**

### Histological and Immunohistochemical assessment

Histological and immunohistochemical investigations were performed on paraffin embedded 3 μm sections using peroxidase-conjugated, affinity-isolated immunoglobulins. All sections were routinely stained with haematoxylin and eosin (H&E) and were processed at the same time to reduce internal staining variations.

Briefly, immunohistochemistry was done according to the instructions of the manufacturer. CD68^+^ macrophages were identified by a 1:50 mouse monoclonal antibody from Dako (Glostrup, Denmark), pre-treatment of the fixed specimen with microwave three times, citrate-buffer pH 6, and as secondary antibody rabbit anti-mouse 1:300 from Dako (Glostrup, Denmark). CD3^+^ T-lymphocytes were identified by a 1:50 polyclonal rabbit antibody from Dako (Hamburg, Germany). Vimentin detection was performed by an anti-vimentin mouse monoclonal antibody 1:100 from Dako, Glostrup, Denmark, pre-treatment of the fixed specimen with microwave three times, citrate-buffer pH 6. Mesothelial cells were detected by a ready to use monoclonal mouse antibody (HBME-1) (Dako, Glostrup, Denmark). COX-2 detection was carried out by a 1:100 rabbit monoclonal antibody from DCS (Hamburg, Germany), pre-treatment microwave three times, citrate-puffer pH 6, and as secondary antibody goat anti-rabbit 1:300 Dako (Glostrup, Denmark). For the detection of C-myc we used a 1:50 rabbit polyclonal antibody from Santa Cruz (California, USA) and as secondary antibody goat anti-rabbit 1:500 (Dako, Glostrup, Denmark). Catenin was analyzed by a ready to use rabbit polyclonal antibody from Spring Bioscience (California, USA) and as secondary antibody goat anti-rabbit 1:500 Dako (Glostrup, Denmark). The expression of immunohistochemical parameters was classified by two independent, blinded observers using a semi-quantitative immunoreactivity score (IRS). Extent of staining was scored as 0 (0-5%), 1 (5-30%), 2 (30-80%) and 3 (80-100%), indicating the percentage of positive stained cells of the area of the section.

Ratio of mesothelial cells to mesenchymal infiltrate was determined from the data of Table 2 and Table 3, with expression of vimentin representing the mesenchymal infiltrate.

**Table 2:** Evaluation of immunohistochemical results of the parietal peritoneum depending on the study groups

	**Day**	**Icodextrin group**	**Sodium chloride group**	**p-value**
**CD68**	7	1.22 ± 0.44	0.81 ± 0.51	0.218
	21	0.78 ± 0.29	0.5 ± 0.5	0.150
**CD3**	7	0.31 ± 0.24	0.19 ± 0.27	0.321
	21	0.17 ± 0.13	0.19 ± 0.17	0.758
**Vimentin**	7	3 ± 0	3 ± 0	1.000
	21	2.31 ± 0.54	2.75 ± 0.66	0.038
**Mesothelial cells**	7	0.53 ± 0.49	0.61 ± 0.76	0.921
	21	2.03 ± 0.88	2.03 ± 0.66	0.689
**Mesothelial cells/Vimentin ratio**	7	0.2 ± 0.2	0.2 ± 0.3	0.921
	21	0.9 ± 0.4	0.7 ± 0.8	0.539
**COX-2**	7	0.19 ± 0.17	0.03 ± 0.08	0.018
	21	0	0	1.000
**C-myc**	7	0	0	1.000
	21	0.14 ± 0.25	0.03 ± 0.09	0.243
**Catenin**	7	0.08 ± 0.13	0.06 ± 0.11	0.609
	21	0.06 ± 0.11	0.03 ± 0.08	0.539

Data shown are mean ± SD.

**Table 3:** Evaluation of immunohistochemical results of the visceral peritoneum depending on the study groups

	**Day**	**Icodextrin group**	**Sodium chloride group**	**p-value**
**CD68**	7	0.7 ± 0.62	0.25 ± 0.25	0.139
	21	0.44 ± 0.54	0.47 ± 0.38	0.748
**CD3**	7	0.03 ± 0.08	0.06 ± 0.11	0.018
	21	0.17 ± 0.18	0.33 ± 0.33	0.738
**Vimentin**	7	2 ± 0.32	2.06 ± 0.39	0.705
	21	1.08 ± 0.22	1.31 ± 0.17	0.028
**Mesothelial cells**	7	1.17 ± 1.02	0.94 ± 1.25	0.293
	21	2.33 ± 0.64	2.11 ± 0.66	0.448
**Mesothelial cells/Vimentin ratio**	7	0.6 ± 0.5	0.5 ± 0.6	0.921
	21	2.2 ± 0.6	1.6 ± 0.4	0.030
**COX-2**	7	0.7 ± 0.62	0.03 ± 0.08	0.152
	21	0.44 ± 0.54	0	1.000
**C-myc**	7	0.03 ± 0,08	0.03 ± 0.08	0.939
	21	0.17 ± 0.18	0.14 ± 0.25	0.457
**Catenin**	7	0.05 ± 0.11	0.08 ± 0.13	0.521
	21	0.06 ± 0.11	0.08 ± 0.13	0.609

Data shown are mean ± SD.

### Statistical analysis

Statistical analysis was carried out using the Statistical Package for Social Sciences software (SPSS®, Vers.17.0, Chicago, IL, USA). Appearance of adhesions was tested with single sided exact Fisher-Test. Differences of the scores between study groups was analyzed by Kruskal-Wallis test for non-parametric data. P-values < 0.05 were considered to be significant. All data are represented as mean ± standard deviation if not otherwise mentioned.

## Results

Two rats died during surgery because of anesthesia complications (one of the icodextrin groups and one of the sodium chloride groups). After surgical intervention all other animals returned to normal activity. None of the rats exhibited local or systemic signs of infection during the observation period.

### Macroscopic evaluation

**Figure 2:**
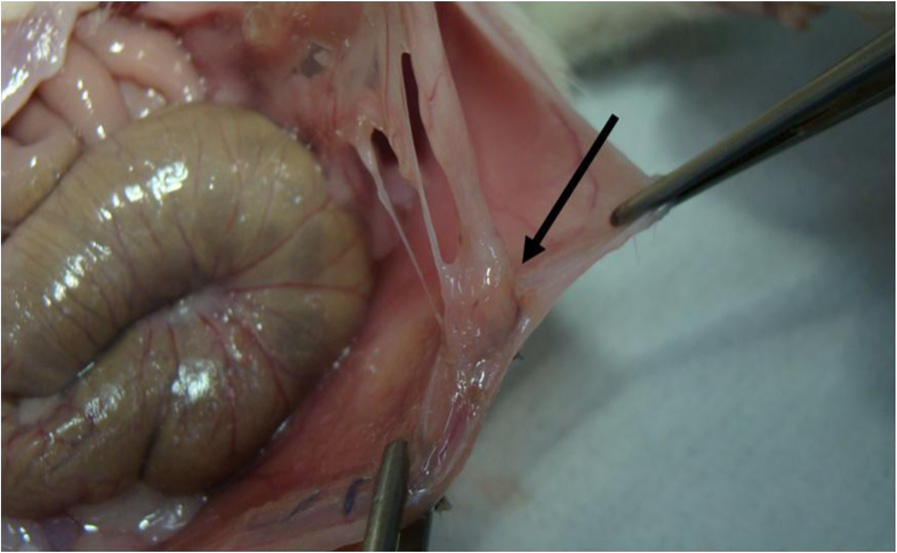
Typical adhesion of the parietal peritoneum after 21 days in the sodium chloride group.

Adhesion formation was prevalent in the sodium chloride group than in the icodextrin group (14/19 (74%) vs. 9/19 (47%); p = 0.048; Figure 2). The remaining animals did not show any signs of adhesions macroscopically. The adhesion score did not reveal significant differences neither between the two study groups nor between the two time periods. Results are shown in Table 4.

**Table 4:** Comparison of macroscopical presence of adhesions and the adhesion score of the icodextrin group and sodium chloride group depending on the time period

	**Icodextrin group**	**Sodium chloride group**	**p-value**
**Presence of adhesions**			
- Day 7	5/10 (50%)	7/10 (70%)	
- Day 21	4/9 (44%)	7/9 (78%)	
- Total	9/19 (47%)	14/19 (74%)	0.048
**Adhesion score**			
- Day 7			
- Visceral peritoneum	2.4 ± 3.4	1.7 ± 2.9	0.544
- Parietal peritoneum	1.6 ± 3.4	2.9 ± 3.2	0.329
- Day 21			
- Visceral peritoneum	2.7 ± 3.4	4.3 ± 2.7	0.292
- Parietal peritoneum	0	1.6 ± 2.4	0.066

Data shown are mean ± SD.

### Microscopic evaluation

After 7 days HE staining revealed an intense formation of adhesions in the area of the peritoneal trauma. The defects were filled up with numerous lymphocytes, plasma cells, macrophages and abundant deposition of collagen. After 21 days these morphologic observations presented with a reduced accumulation of these mononuclear cells but increased deposition of collagen.

### Immunohistochemical observations

The immunohistochemical observations were determined separately for visceral and peritoneal peritoneum. All findings were evaluated and compared with respect to time and active agent.

#### Parietal peritoneum

The immunohistochemical findings of the parietal peritoneum are shown in Table 2. In evaluation of CD68 as a marker of macrophages and monocytes we saw more CD68^+^ cells in the icodextrin group than in the sodium chloride group, however not reaching significance. Over time the expression decreased significantly in the icodextrin group from day 7 to day 21 (p = 0.021), but not in the sodium chloride group.

CD3^+^ T-lymphocytes were constantly seen in both groups at both time points. Vimentin as a marker of cell integrity was expressed significantly lower after 21 days than after 7 days in the idodextrin group (p = 0.002), being even significantly lower than in the sodium chloride group at day 21 (p = 0.038).

After 21 days the detection of mesothelial cells showed a significant elevation in both the icodextrin and the sodium chloride group (icodextrin group: p = 0.004; sodium chloride group: p = 0.003), without significant differences in comparison of the applied agents.

Expression of COX-2 was quite rare in both groups. However it was significantly higher in the icodextrin group at day 7 (p = 0.018). After 21 days there was no longer a positive expression of COX-2 in either group.

Expression of C-myc was negative in both groups at day 7, though at day 21 there were some positive findings in both groups without any statistical difference.

#### Visceral peritoneum

The immunohistochemical findings of the visceral peritoneum are shown in Table 3. Macrophages were distributed equally in both groups and at both time points. Again, the expression of CD3^+^ T-lymphocytes was generally comparatively low, however, a significant elevation of CD3^+^ T-lymphocytes over the time was observed in the sodium chloride group but not in the icodextrin group (p = 0.017, and p = 0.471, respectively).

Vimentin as a marker of cell integrity was expressed significantly lower after 21 days than after 7 days in both groups (each p < 0.001). After 21 days the expression was lower in the icodextrin group than in the sodium chloride group (p = 0.028).

The detection of mesothelial cells as indicator for reperitonealisation showed a significant elevation in both groups over the time (icodextrin group: p = 0.021; sodium chloride group: p = 0.011). The ratio of mesothelial cells to mesenchymal infiltrate (vimentin) was significantly elevated in the icodextrin group in comparison to the sodium chloride group in (2.2 ± 0.6 vs. 1.6 ± 0.4; p = 0.030).

We observed higher values of COX-2 in the icodextrin group after 7 days with a marked decrease at day 21 (p = 0.039).

Whereas catenin was rarely expressed in both groups at both time points, the expression of C-myc showed a slight increase over time in both study groups, reaching significance for icodextrin (p = 0.035).

## Discussion

Injuries to the peritoneum are an inevitable occurrence during surgery, with an accompanying healing process which frequently results in the adherence of adjacent organs by a fibrous mass, commonly denoted as adhesions [5,21]. One attempt to reduce adhesion formation is the application of bio-absorbable mechanical substances like icodextrin in order to avoid the agglutination of the affected organs [4].

Several studies showed a significant reduction of adhesion formation after abdominal surgery due to the application of icodextrin [2]. Rodent models are an established experimental setting in order to simulate human adhesion formation [18,22]. Aim of this study was to show the influence of icodextrin on tissue response, adhesion formation and wound healing, of the parietal and visceral fraction in a rat model.

Some studies describe a significantly positive influence of icodextrin on the prevention of adhesion formation others do not [23,24]. A recent prospective randomized controlled trial of *Catena et al.* showed that the use of icodextrin in adhesive small bowel obstruction is safe and reduces intra-abdominal adhesion formation and the risk of re-obstruction [9].

The current study indicates that icodextrin may function rather by altering the local repair process than by acting as a surface barrier. We observed less adhesion in the peritoneal defect. At both sites the initially enhanced accumulation of inflammatory and mesenchymal cells in the icodextrin group almost disappeared over time. This resulted in an improved ratio of mesothelial cells to vimentin expression indicating an enhanced reperitonealisation with a reduced mesenchymal scar infiltrate.

Icodextrin might not serve as a simple physico-chemical barrier just reducing cell activation and invasion. It might stimulate the local inflammatory defense, and thereby may trigger the local immunological response resulting in less scar formation. In this regard our findings suggest the importance of the initial adaptive immune response to a trauma for the later balance of scarring and re-peritonealisation or regeneration. These results provide further options to prevent adhesions by modifying the immune response instead of just blocking it.

The identification of macrophages (CD68) and T lymphocytes (CD 3) proves an inflammatory activity which is in accordance with the findings of *Binnebösel et al.* who found persistent inflammatory activity in adhesions even years after initial surgery [16,18]. *Hoshino et al.* analyzed the importance of the activation of peritoneal macrophages and their expression of chemokine receptors in promoting post-operative and post-inflammatory peritoneal adhesion formation [25].

In many studies it has been shown that COX-2 reflects the inflammatory activity normalizing within weeks after injury [26-28]. Similarly, we found a close correlation between appearance of macrophages and expression of COX-2. We observed low COX-2 expression indicating a deactivation of the inflammatory situation.

It is generally accepted that vimentin is the major cytoskeletal component responsible for sustaining cell integrity, especially found in connective tissue and smooth muscles [29]. *Gonzales et al.* demonstrated its crucial influence on matrix adhesion in endothelial cells [30]. *Nieminen et al.* described that vimentin is required in both the receiving endothelial cell and mononuclear cell to stabilize endothelial cell interactions [31]. The decrease of vimentin after 21 days in the study groups might indicate the incipient reconstitution of cell integrity of the peritoneum. Interestingly, the expression of vimentin was significantly higher in the parietal peritoneum than in the visceral peritoneum in both study groups at both points of time. These findings suggest that repair mechanisms and restoration of cell integrity in the parietal peritoneum differs from the visceral peritoneum. Vimentin as a general marker of mesenchymal cells may reflect the formation of any scar tissue with fibroblasts, endothelial cells and smooth muscle cells [30]. Thus a higher ratio of mesothelial cell/vimentin expression might indicate an improved wound repair. The mesothelial cell layer functions as a natural barrier between the peritoneal cavity and the adjacent connective tissue [32]. Previous studies demonstrated that the transplantation of mesothelial cells using intraperitoneal injection is effective for the prevention of peritoneal adhesions [33,34]. We found significantly elevated levels of the expression of mesothelial cells after 21 days in all study groups indicating the regeneration process of both visceral and parietal peritoneum.

The C-myc and catenin pathways are known for their important role in tissue remodelling, wound healing and regeneration [35,36]. The low expression in our experimental setting might indicate that the C-myc and catenin pathways are not appropriate markers in order to illustrate peritoneal repair mechanisms.

Overall, icodextrin did not induce any signs of persisting elevated inflammatory reaction in comparison to the sodium chloride group indicating its excellent biocompatibility. Although the adhesion score was not significantly lower after icodextrin treatment, our study revealed a significantly lower presence of adhesion formation in the icodextrin group than in the sodium chloride group supporting the theory that icodextrin is capable of reducing adhesion formation, even though detailed cellular mechanisms are still not comprehended. One major limitation of our study is the absence of a control group without peritoneal abrasion or parietal defect to establish normal values for several immunohistochemical parameters. However, the intention was to simulate repair mechanisms of the visceral and parietal peritoneum with and without the presence of icodextrin. Furthermore, conclusions have to be drawn with caution since results from rodent models cannot be directly translated to the situation in humans. Further clinical studies have to demonstrate the definitive effect of icodextrin on regeneration mechanisms of the visceral and parietal peritoneum.

## Conclusions

The intraperitoneal application of 4% icodextrin reduces adhesion formation in comparison to sodium chloride. 4% icodextrin solution reduces the inflammatory and mesenchymal infiltrate in the wounded area, improving the ratio of mesothel cells to mesenchymal infiltrate. As here demonstrated, icodextrin is able to ameliorate the local tissue response. Further experimental studies would elaborate the impact on the early response of the adaptive immune system, which then may trigger the subsequent wound healing and tissue repair.

## Competing interests

The authors declare that they have no competing interests.

## Authors’ contributions

CDK and MB made substantial contributions to the conception and design of the study. MB and RR critically revised the manuscript for important intellectual content. PS contributed to acquisition of data. CDK performed the statistical analysis. SJ, RT and UK were involved in analysis and interpretation of data. UPN gave final approval of the version to be published. All authors read and approved the final manuscript.

## Pre-publication history

The pre-publication history for this paper can be accessed here:

http://www.biomedcentral.com/1471-2482/13/34/prepub
